# FDA-Cleared Artificial Intelligence Medical Devices in Orthopaedic Surgery

**DOI:** 10.5435/JAAOSGlobal-D-25-00170

**Published:** 2026-02-05

**Authors:** Branden Lee, Mitchell Jay, Henry Fox, James Padley, Tinglong Dai, Adam S. Levin

**Affiliations:** From the Department of Orthopaedic Surgery, Johns Hopkins Medicine (Mr. Lee, Dr. Fox, Dr. Padley, and Dr. Levin); School of Medicine, Johns Hopkins University (Mr. Lee and Mr. Jay); Carey Business School, Johns Hopkins University, Baltimore, MD (Dr. Dai); Hopkins Business of Health Initiative, Johns Hopkins University, Washington, DC (Dr. Dai), and School of Nursing, Johns Hopkins University, Baltimore, MD (Dr. Dai).

## Abstract

**Background:**

Artificial intelligence (AI) and machine learning are powerful computational approaches that have the capacity to automate and improve medical care delivery in orthopaedic surgery through augmentation of medical devices, from diagnostic modalities to surgical guidance. Existing research has focused on prospective device applications and ongoing clinical trials, but a comprehensive analysis on cleared devices by the FDA is lacking.

**Methods:**

A retrospective analysis was conducted for 70 FDA-cleared AI/machine learning–based medical devices (AIMDs) for orthopaedic surgery indications as of February 2025. These devices were categorized by indicated use, corresponding orthopaedic subspecialty, development history, AI architecture, and commercialization approach. For commercialization approach, active manufacturers were categorized by private or publicly traded status, acquisition history, and headquartered country.

**Results:**

Since the first orthopaedic AIMD clearance in 2017, the 3-year moving average of AIMD clearances increased from 3.0 devices/year from 2017 to 2019 to 16.6 from 2022 to 2024. Alongside this growth, deep learning emerged as the dominant AI technique, comprising 57.3% of AIMDs approved from 2022 to 2024. Spine surgery was the most common orthopaedic subspecialty for devices, representing 42.9% of devices, followed by hip and knee at 20.0%. Surgical planning predominated across orthopaedic subspecialties except in trauma, where devices focused on fracture identification and surgical guidance. 62.2% of orthopaedic AIMDs cleared from 2017 to 2019 lacked any clinical testing, but this rate declined to 19.7% from 2022 to 2024. Overall, 22.8% of orthopaedic AIMDs lacked clinical testing and 68.6% were tested with retrospective data sets. Only 8.6% were validated through a formal, prospective clinical trial.

**Conclusion:**

Although AI represents an exciting and rapidly developing area of innovation in orthopaedic surgery, improved regulatory safeguards and clinical evaluation standards are essential for the evidenced adoption and safe implementation of these promising technologies.

Artificial intelligence (AI) and machine learning (ML) is an emerging computational framework and platform that has the potential to revolutionize medical care delivery. From facilitating automation to enhancing guidance systems, AI and ML can transform the scale and scope of medical devices. This is particularly relevant for a field like orthopaedic surgery that is rife with innovation in the medical device arena.^1-3^ Existing literature on the application of AI in orthopaedic surgery typically analyzes case-use AI/ML applications^4,5^ or focuses on prospective approaches,^6^ which leaves a critical gap in understanding the characteristics, development trends, and clinical adoption of AI/ML-enabled medical devices (AIMDs) that have already been cleared by the FDA.

A total of 1,017 AIMDs have already been cleared by the FDA, with an exponential growth of new devices in the past 3 years.^7^ Over 95% of AIMDs are reviewed by the FDA through the 510(k) pathway, which allows for clearances of devices through demonstrations of substantial equivalence to other devices on the market.^8,9^ The accelerated growth of device applications, coupled with the reduced threshold of substantial equivalence to attain device clearance, has led to several concerns about the evaluation of these new devices.^10^ Several evidentiary gaps for cleared AIMDs have already been reported, which includes the finding that less than 5% of cleared devices report conducting a clinical trial.^7,9^

In addition, nearly 10% of AIMDs have been subject to device recalls,^8^ highlighting some of the safety risks associated with the current regulatory infrastructure. Analysis of FDA-cleared AIMDs across commercialization approaches has not only highlighted significant differences in device characteristics but also showed that these recall rates have been driven by publicly traded companies, which possess >30-fold greater device recall incidences compared with privately held companies. The extent of these issues, the commercialization landscape, and the characteristics of FDA-cleared AIMDs specific to orthopaedic surgery have yet to be reported.

The primary purpose of this study was to identify AIMDs cleared by the FDA for use in orthopaedic surgery and to evaluate the trends and characteristics of these devices, including indicated clinical function, orthopaedic subspecialty focus, AI architecture, device recall or adverse event history, and the extent of clinical testing that led to device clearance. The second purpose was to investigate the commercial landscape of cleared devices by assessing the size and types, headquartered region, and commercialization strategy (acquired versus in-house developed) of companies actively manufacturing devices. The hypothesis was that AIMDs for orthopaedic surgery would be rising in prevalence, with variations in device characteristics and commercialization landscape changing over time.

## Methods

This analysis was exempt from Institutional Review Board approval because it did not involve human participant research. This study adhered to the Strengthening the Reporting of Observational Studies in Epidemiology reporting guideline for cohort studies.^11^

### Data and Study Sample

A cohort of 1,017 FDA-cleared AIMDs was identified using the FDA-cleared AI/ML-Enabled Medical Device Database as of February 15, 2025. From these devices, orthopaedic-specific devices were identified by the following inclusion criteria: (1) if diagnostic (including radiographic analysis)—the device specifically and exclusively targeted the musculoskeletal systems and (2) if surgical guidance—the device explicitly mentioned orthopaedic procedures. This approach included all spine surgery-specific devices. This approach was chosen to identify devices specifically developed for orthopaedic procedures, rather than general surgery-supportive devices that could be used in orthopaedic surgery.

For each AIMD, the filing company name and country, clearance year, route of regulatory review, recall status, regulatory codes, device description, adverse event reports, AI approach, and clinical service were obtained from the FDA-approved AI/ML-Enabled Medical Device, 510(k), de novo Approval, Manufacturer and User Facility Device Experience Database, and Medical Device Recall Databases. Information pertinent to the AIMD manufacturing companies—including headquarter location, company status (eg, subsidiary, parent, or active), merger and acquisition history, and market capitalization of public companies was collected from the S&P Capital IQ NetAdvantage and PrivCo databases. These databases are established financial resources for publicly traded and privately held companies, respectively. Company status and any acquisitions were verified by company legal notices where applicable.

### Defining Device Development-Commercialization Models

The device originator in the FDA application was assessed using the NetAdvantage and PrivCo databases, along with the legal terms of service for each originator website, to determine (1) whether the filing company was a subsidiary of a larger company and (2) whether the company had undergone any significant acquisitions. In cases of acquisitions, the acquiring company was identified as the active entity for the device and the device's acquired status was recorded.

Each active manufacturer was classified as a publicly traded company, private company, academic/public organization, or defunct (out of business). Public companies were further categorized by market capitalization thresholds: mega (>$200 billion), large (<$200 billion but >$10 billion), mid (<$10 billion but >$2 billion), and small (<$2 billion). Devices were grouped into six different commercialization models based on these manufacturer classifications and device attainment (acquired versus developed in-house) using a previously published approach.^12^

### Artificial Intelligence Model Architecture

FDA device summary review documents were analyzed to identify whether an AI-specific or AI-exclusive approach was explicitly stated, which was inclusive of devices with FDA product codes that guarantee AI or ML use: QIH, QJU, QDQ. The FDA QIH code refers to “automated radiological image processing software,” QJU refers to “image acquisition and/or optimization guided by artificial intelligence,” and QDQ refers to “radiological computer assisted detection/diagnosis software for lesions suspicious for cancer.” Although all devices included in this cohort and the FDA database have been verified to use AI by the FDA, this variable was analyzed to assess the transparency of software approach of these devices.

Generic terms such as “automate,” “segment,” or “algorithm,” which may not necessarily indicate AI, were considered nonexplicit since non-AI computational approaches could perform these functions. If a device had an identical design to a previous version, the AI architecture of the predicate device was applied. Devices were classified as employing multiple AI approaches only when distinct AI methods were explicitly declared for separate functions (eg, computer vision for image analysis and ML for patient risk stratification).

### Device Function, Subspecialty, and Clinical Testing

Orthopaedic-specific medical devices were categorized by their subspecialty based on the indicated function of the device. Subspecialties included spine, hip, or knee (includes complete leg length devices); general (multiple or generalized musculoskeletal indications); foot or ankle, shoulder, trauma (includes all devices pertinent to rib fractures); and hand or wrist (includes all upper extremity devices not specific to the shoulder.)

Devices were also categorized by functionality of devices-surgery planning, fracture identification, and surgical guidance. Surgery planning devices include all preoperative and diagnostic radiographic modalities and analysis, excluding those with a bone fracture-specific functionality. Surgical guidance devices include all devices with indications for intraoperative uses.

Finally, devices were analyzed according to their clinical validation approach using previously published approaches.^12^ Reported device testing was defined into one of three categories: (1) no clinical validation, relying on bench testing of device software; (2) retrospective clinical testing, relying on existing clinical data repositories; and (3) prospective clinical testing, relying on prospective recruitment of patients for device validation. We also extracted the sample size of each validation study to assess the clinical power of these studies.

### Analysis

Descriptive statistics (mean, SD, etc) were calculated for the number and proportion of devices across orthopaedic subspecialties, reported AI architectures, device function, reported clinical testing of medical device, and commercialization approach. To address year-to-year variations, time trends were analyzed using 3-year moving averages (2017 to 2024) for consecutive years. Simple linear regression, along with R^2^ and 95% confidence intervals, was calculated when appropriate. All analyses and visualizations were conducted using GraphPad Prism version 10.0.3 (GraphPad Software).

## Results

### Trends in FDA Clearances of Artificial Intelligence/Machine Learning–Based Medical Devices for Orthopaedic Surgery

**Figure 1:**
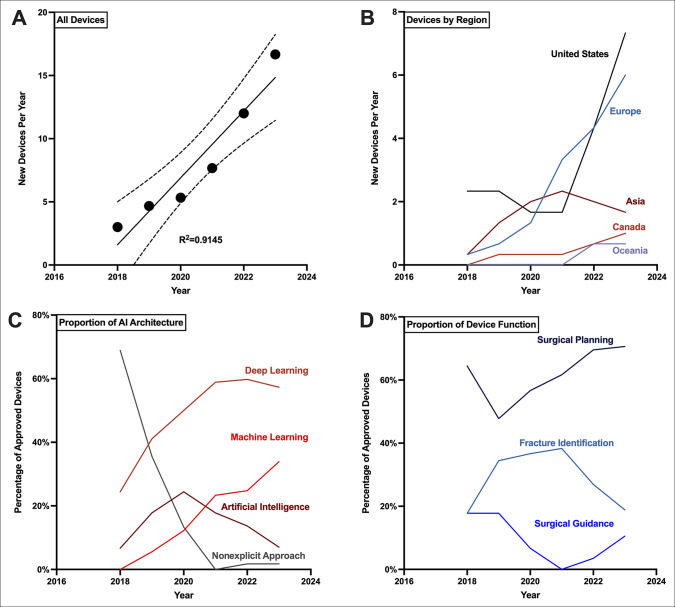
Graph showing 3-year moving average trends of FDA-cleared orthopaedic surgery AI medical devices, 2017 to 2024. Three-year moving averages from 2017 to 2024 were calculated and plotted at the 3-year center year for (**A**) number of all new devices annually, (**B**) number of new devices contributed annually by each geographic region, (**C**) annual proportion of AI architecture used for new devices, and (**D**) annual proportion of clinical indication for new devices. Simple linear regression was calculated for all devices by their year of clearance, which is shown as a solid black line. 95% confidence intervals are shown by the dashed lines and the R^2^ value is listed. AI = artificial intelligence

Since 2017, there have been 70 total AIMDs relevant to orthopaedic surgery that have been cleared by the FDA. Figure 1 illustrates the yearly trends in clearances for these medical devices. Cleared orthopaedic surgery AIMDs have grown consistently, demonstrating a linear relationship between the 3-year moving average of new AIMDs and year of clearance (R^2^ = 0.9114, Figure 1, A). This explains a >5.5-fold increase in new devices, from an average of 3.0 clearances annually between 2017 and 2019 to 16.6 between 2022 and 2024.

Many of these devices are commercialized by US companies (44.3%), which have demonstrated a growing share of new devices (Figure 1, B). Between 2022 and 2024, US companies accounted for 22 cleared devices (44.0% of new devices in this period). However, European companies have taken a competing and large role, from one device clearance between 2017 and 2019 (11.1% of new devices in this period) to 18 devices between 2022 and 2024 (36.0% of new devices in this period). This is followed by Asian (10.0% of new devices between 2022 and 2024), Canadian (6.0% of new devices between 2022 and 2024), and Oceanian (4.0% of new devices between 2022 and 2024) companies.

When considering AI architecture represented by new devices, ML and deep learning (DL) techniques have seen growing roles (Figure 1, C). Between 2017 and 2019, no devices used non-DL ML techniques and 24.4% reported DL architectures. This grew to 33.9% for non-DL ML utilization and 57.3% for DL utilization between 2022 and 2024. Non-ML/DL AI utilization rose from 6.7% between 2017 and 2019 to a peak of 24.4% between 2019 and 2021, but eventually returned to 7.0% between 2022 and 2024. Notably, rates of non-explication of any AI utilization fell from 68.9% between 2017 and 2019 to 1.8% between 2022 and 2024.

Similarly, device applications changed over time (Figure 1, D). Surgical planning devices remained the leading application of new AIMDs, representing 64.4% of new devices between 2017 and 2019 and up to 70.6% between 2022 and 2024. Fracture identification represented 17.8% of new devices between 2017 and 2019, which grew to >30% for the next three 3-year periods. However, this returned to 18.9% between 2022 and 2024. Conversely, the use of surgical guidance devices fell from 17.8% in 2017 to 2019 to 0% between 2018 and 2020. This returned to 10.5% of new devices between 2022 and 2024.

### Device Characteristics Across Orthopaedic Subspecialties

**Figure 2:**
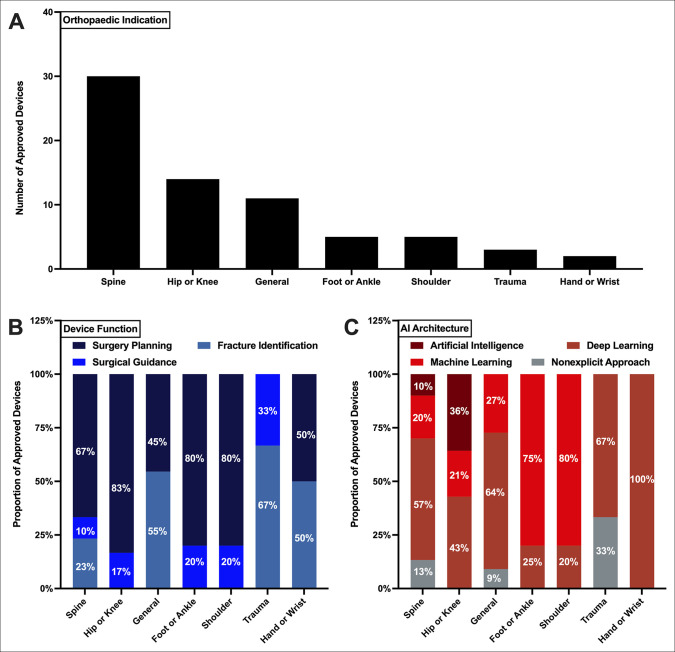
Graph showing AI medical device characteristics across indicated orthopaedic subspecialties. **A**, Total number of FDA-cleared devices across each orthopaedic subspecialty. Proportion of reported (**B**) device function and (**C**) AI architecture for FDA-cleared devices within each orthopaedic subspecialty is shown. AI = artificial intelligence

Of the orthopaedic subspecialties, AIMDs for spine surgeries were the leading indication, representing 30 total devices (42.9%, Figure 2, A). This is followed by hip or knee (20.0% of devices), general orthopaedic (15.7%), foot or ankle (7.1%), shoulder (7.1%), trauma (4.3%), and hand or wrist (2.9%). Overall, surgical planning is the leading function across most surgical subspecialties—67% of spine devices, 83% of hip or knee, 80% of foot or ankle, 80% of shoulder, and 50% of hand or knee devices (Figure 2, B). Fracture identification was a common application for general orthopaedic surgical (55%), trauma (67%), and hand or wrist (50%) devices. Surgical guidance devices were less common overall but represented 20% of foot or ankle, 20% of shoulder, 33% of trauma, 17% of hip or knee, and 10% of spine surgery devices.

As the leading architecture of the cohort, DL utilization was the most common application for many subspecialties (Figure 2, C), including spine (57%), hip or knee (43%), general orthopaedic (64%), trauma (67%), and hand or wrist (100%). ML was the leading architecture for foot or ankle (80%) and shoulder (80%) devices. General AI architecture was not used for any devices in most orthopaedic subspecialties, with the exception of hip or knee (36%) and spine (10%) devices. Nonexplication of AI use was relatively uncommon across subspecialties, but represented 33% of trauma, 13% of general, and 9% of spine devices.

### Clinical Testing and Safety of Medical Devices

**Figure 3:**
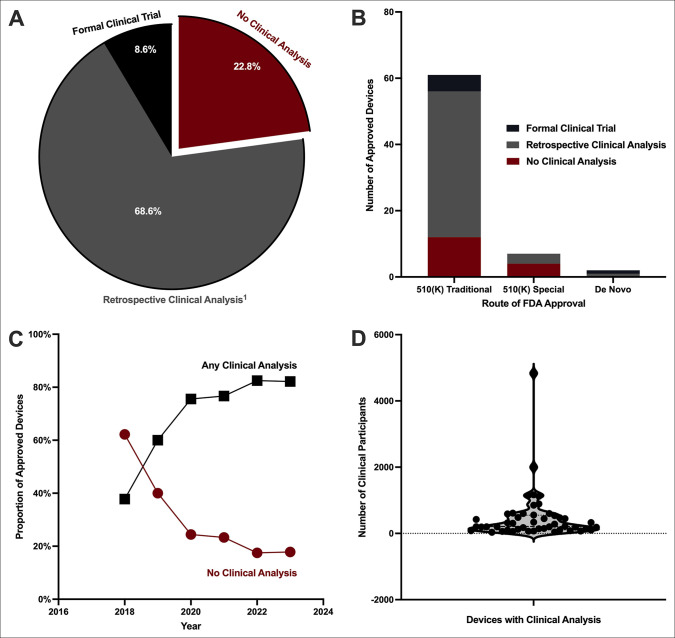
Graph showing clinical testing reported for orthopaedic artificial intelligence medical devices. **A**, Proportion of devices with formal prospective clinical trials, retrospective clinical analysis, or no clinical analysis reported. **B**, Number of devices with each level of reported clinical testing across pursued regulatory pathways. **C**, Three-year moving average of cleared devices with any or no clinical analysis reported. **D**, Violin plot demonstrating the number of clinical participants analyzed for clinical testing. Each dot represents a unique clearance. Six of these devices reported retrospective clinical image testing, but did not report the number of clinical participants evaluated and were not included in the violin plot but otherwise included in the figure.

Beyond AI model training, many device applications reported additional clinical testing, with 8.6% reporting a formal clinical trial and 68.6% reporting a retrospective clinical analysis (Figure 3, A). However, 22.8% reported no clinical testing, relying on technical or clinical similarities to predicate device for device clearance. This is particularly evident for the seven devices pursuing a 510(K) special review, with 57.1% of these devices reporting no clinical testing and 42.9% of these devices reporting a retrospective testing using clinical imaging repositories (Figure 3, B). Of the 61 devices that underwent a conventional 510(k) review, 19.7% did not conduct any clinical testing, 72.1% reported a retrospective clinical analysis, and 8.2% reported a formal prospective clinical trial.

Of the 44 devices cleared through a conventional 510(k) pathway with reports of a retrospective clinical analysis, 13.6% failed to report the number of clinical participants included in their analysis. As expected, both of the devices approved through the de novo regulatory process reported clinical testing, which included a device with a formal prospective clinical trial and another with a retrospective clinical analysis. One of these *de novo*-approved devices, *Rho*, was the only device in the cohort to receive a breakthrough device designation.

Over the years, the use of clinical analysis has improved (Figure 3, C). Between 2017 and 2019, the 3-year moving average proportion of devices without any clinical analysis reported was 62.2%, which fell to 17.8% between 2022 and 2024. Between these two respective time periods, the 3-year moving average proportion of cleared devices with clinical analysis grew from 37.8% to 82.2%. Furthermore, of the six devices reporting a formal prospective clinical trial, five were cleared between 2022 and 2024, with four of these in 2024 alone.

However, the number of clinical participants evaluated within each clinical analysis showed wide variability (Figure 3, D). The mean number of clinical participants evaluated per device is 469.4 (SD, 747.9), with a median of 208.0 participants per device (IQR, 107.3 to 550.8). Notably, despite the lack of clinical testing in many devices and variability in power of clinical analysis, no orthopaedic AIMD has been subject to a device recall or medical device report of adverse events.

### Commercialization of Artificial Intelligence Medical Devices in Orthopaedic Surgery

**Figure 4:**
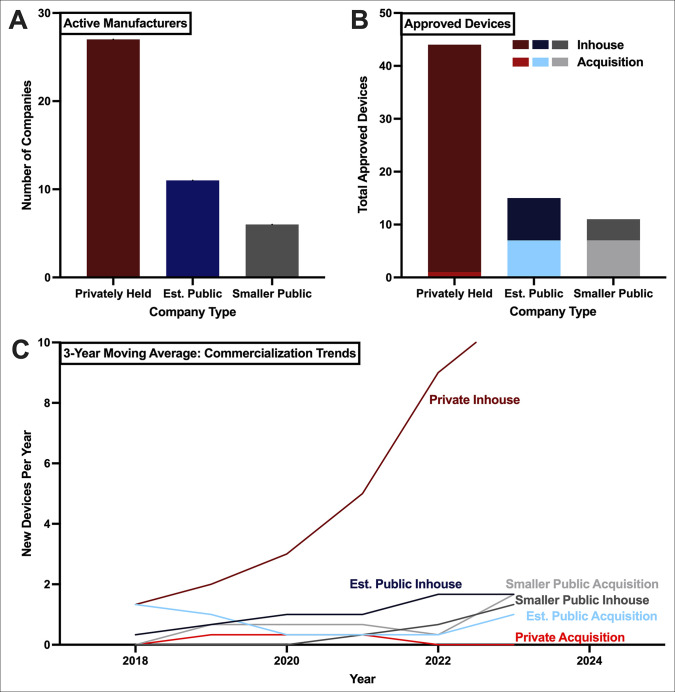
Graph showing commercialization landscape of orthopaedic artificial intelligence medical devices. **A**, The number of companies actively manufacturing artificial intelligence medical devices for orthopaedic surgery is shown for each company type. **B**, The number of devices across these company types is shown characterized by whether the device was acquired or developed in-house. **C**, Three-year moving average between 2017 to 2024 was calculated and plotted at the 3-year center for number of new devices by commercialization model.

There are 44 total companies actively manufacturing FDA-cleared AIMDs, of which 27 (61.3%) are privately held, 11 (25.0%) are established public companies, and 6 (13.6%) are smaller public companies (Figure 4, A). Device share is comparable with these rates, with private companies contributing 44 (62.8%) FDA-cleared AIMDs, 15 (21.4%) by established public companies, and 11 (15.7%) by smaller public companies (Figure 4, B). However, there are large discrepancies in commercialization models, as nearly all devices contributed by private companies are developed internally by the company (97.8%). These rates are contrasted to internal device development rates of 53.3% for established public companies and 36.4% for smaller public companies, with both company types demonstrating stronger tendencies toward a device acquisition strategy, as opposed to internal device development.

When considering these commercialization approaches over time, it is evident that private internal development of AIMDs outpaces any other commercialization approach (Figure 4, C). Between 2022 and 2024, the 3-year moving average number of internally developed devices contributed by private company (11.0 devices/year) is nearly double the contribution of every other model combined, which includes established public development and smaller public acquisition (both 1.7 devices/year), smaller public development (1.3 devices/year), and established public acquisition (1.0 devices/year).

## Discussion

The increasing integration of AI in orthopaedic surgery has driven a paradigm shift in surgical planning, diagnostic accuracy, and patient-specific interventions. This study presents a comprehensive analysis of FDA-cleared AIMDs specific to orthopaedic surgery, highlighting their clearance trends, clinical applications, and commercialization pathways. Our findings suggest that while approval of AI devices continues to expand the influence of AI in orthopaedic surgery, key challenges remain regarding regulatory oversight, clinical validation, and adoption into practice.

The primary finding of this study is that the annual rate of FDA clearances for AIMDs in orthopaedic surgery has consistently increased from 2017 to 2024. The 3-year moving average of annual clearances increased 5.5-fold in this span, showing comparable growth to the 3.8-fold increase between 2017 and 2020 reported by Zhu et al^13^ and the 5.3-fold increase between 2017 and 2022 reported by Joshi et al.^9^ All orthopaedic AIMDs, as either hardware or software, used AI to augment analysis of radiographic imaging, consistent with previous research reporting radiographic interpretations as the leading application of all AIMDs.^8,9^ While surgical planning was the leading application for these radiographic analyses, fracture identification and surgical guidance technologies represented sizeable device shares. These applications represent the growing applications of AI within important and identified areas of orthopaedic innovation.^14-16^ However, with this growth catalyzed by a globalized distribution of AIMD manufacturers, these results highlight an important scaffold of harmonized regulation standards and deployment or access of these new devices.^17,18^

Another important finding of our study was spinal and hip/knee reconstruction were the most represented subspecialties in the FDA-cleared AIMDS, representing 62.9% of all cleared devices. This finding is consistent with previous predictions of AIMD roles in orthopaedic contexts. Spine surgeries were predicted to have a very high number of use cases, because of the high surgical risks and surgical complexities.^19,20^ Total joints also ranked highly, stemming from existing robotic architecture that may be repurposed and improved with AI augmentation.^20^ Surgical planning devices predominated across all orthopaedic services besides trauma, which is afforded limited time for surgical planning relative to elective subspecialties and may instead benefit from prompt fracture identification and/or urgent surgical guidance technologies.^20,21^ A small number of devices were approved in the shoulder, foot and ankle, trauma, and hand/wrist spheres, which may help guide future AI-related endeavors and innovation.

The patterns of orthopaedic AIMD commercialization may also be instructive in guiding future research, development, and business decisions. The commercialization of orthopaedic AIMDs is predominantly driven by private companies, which account for >60% of active manufacturers and new devices. These private companies primarily focus on in-house development of new devices, while publicly traded firms, both smaller and established, were more likely to acquire their AI technologies. By contrast, we previously showed that (1) privately held companies demonstrate a comparable device-share to publicly traded companies in the commercialization of all AIMDs and (2) both public and private companies were more likely to develop devices in-house.^12^ The stronger acquisition-driven commercialization by established firms for orthopaedic devices warrants further investigation, particularly in determining how corporate consolidation affects device quality, regulatory compliance, and clinical accessibility.

Another notable result from our study involves the characterization of clinical testing data associated with these new devices. Over three-quarters of cleared orthopaedic AIMDs reported some form of clinical validation studies, although only 8.6% report formal, prospective clinical trials. This is a considerable improvement on rates reported for all AIMDs, with only 70.4% reporting any preperformance studies^7^ and 3.2% reporting a clinical trial.^9^ In addition, the rates of orthopaedic AIMDs reporting no clinical data improved from 62.2% to 17.8% between the first and last 3 years of the study period. Furthermore, there were no reported recalls associated with any of the cleared orthopaedic AIMDs. Although these devices have a short postclearance time at the time of analysis, these rates are contrasted with the 10% recall rate for all AIMDs.^8^ The lack of recalls also contrasts to the 17.8% recall rate of non–AI-enabled orthopaedic devices cleared through the 510(k) pathway.^22^ Notably, orthopaedic medical devices have been reported to contribute between 11.8% and 21.5% of all medical device recalls.^23^

Although our results show a relative improvement in recall rates and premarket validation for orthopaedic AIMDs compared with the recall rates of all AIMDs (and those of non–AI-related orthopaedic medical devices), the objectively low rate of prospective clinical trials and large variance in clinical testing participants in validation studies underscores an opportunity for improved clinical testing. Although there is significant variability in these device applications and subsequently the requirements for validation, many device clearance summaries preclude insight into necessary variables for quality assessments of these studies, including demographics, randomization/sampling approach, and raw outcome measures. These are necessary considering the long-standing concerns of AIMD reporting transparency, rigor of premarket device validations, and generalizability of reported findings.^7,12,24^

There are several limitations to this study. First, the analysis relies on FDA-cleared AIMDs, which does not capture devices in earlier investigational phase, currently under regulatory review, or approved in other countries. This limits the global generalizability of our findings and potentially excludes upcoming advancements in new devices. Furthermore, this database is limited to the retrospective inclusion of cleared devices provided by the FDA, potentially excluding some newly cleared devices. Second, the analysis was conducted on devices with orthopaedic-specific indications, potentially excluding other intraoperative AIMDs that are not specific to, but could be used in orthopaedic surgery. This approach was chosen to specifically focus on AI innovation efforts specifically and exclusively in orthopaedics, but this provides limited insight into general AI applications that could be applied to surgical subspecialties like orthopaedic surgery. Third, this study does not capture or evaluate real-world clinical performance, surgeon adoption rates, or long-term patient outcomes. Future research should focus on postmarket surveillance and clinician-reported experiences to provide further context on the adoption and reliance on these emerging technologies.

## Conclusion

Driven largely by innovation of private companies, an increased number of AIMDs spanning multiple subspecialties and functionalities have been cleared for use in orthopaedic surgery. However, this innovation should also be balanced with improved regulatory safeguards, including mandatory public reporting of all clinical validation results, requirements for diverse clinical testing participants, and continued postmarket surveillance testing.
